# Prognostic value of programmed cell death ligand 1 (PD-L1) expression in patients with stage III non-small cell lung cancer under different treatment types: a retrospective study

**DOI:** 10.31744/einstein_journal/2024AO0575

**Published:** 2024-06-05

**Authors:** Nicoly Marques de Castro, Fernando Moura, Aline Lury Hada, Diogo Garcia, Elivane da Silva Victor, Gustavo Schvartsman, Leonardo Carvalho, Milena Lourenço Coleta Fernandes, Rodrigo de Souza Martins, Elaine Ferreira da Silva, Sarah Silva Mello Batista dos Santos, Letícia Taniwaki, Patrícia Taranto, Janaina Pontes, Juliana Rodrigues Beal, Ana Carolina Pereira Dutra, João Bosco de Oliveira, Sérgio Eduardo Alonso Araujo, Pedro Luiz Serrano Usón

**Affiliations:** 1 Hospital Israelita Albert Einstein São Paulo SP Brazil Hospital Israelita Albert Einstein, São Paulo, SP, Brazil.

**Keywords:** Carcinoma, non-small-cell lung cancer, Lung neoplasms, Adenocarcinoma, Programmed cell death 1 receptor

## Abstract

**Objective:**

Currently programmed cell death protein 1 (PD-1) inhibitors in combination with other therapies are being evaluated to determine their efficacy in cancer treatment. However, the effect of PD-ligand (L) 1 expression on disease outcomes in stage III (EC III) non-small cell lung cancer is not completely understood. Therefore, this study aimed to assess the influence of PD-L1 expression on the outcomes of EC III non-small cell lung cancer.

**Methods:**

This study was conducted on patients diagnosed with EC III non-small cell lung cancer who underwent treatment at a tertiary care hospital. PD-L1 expression was determined using immunohistochemical staining, all patients expressed PD-L1. Survival was estimated using the Kaplan-Meier method. Relationships between variables were assessed using Cox proportional regression models.

**Results:**

A total of 49 patients (median age=69 years) with EC III non-small cell lung cancer and PD-L1 expression were evaluated. More than half of the patients were men, and most were regular smokers. The patients were treated with neoadjuvant chemotherapy, surgery, or sequential or combined chemotherapy and radiotherapy. The median progression-free survival of the entire cohort was 14.2 months, and the median overall survival was 20 months. There was no significant association between PD-L1 expression and disease progression, clinical characteristics, or overall survival.

**Conclusions:**

PD-L1 expression was not correlated with EC III non-small cell lung cancer outcomes. Whether these findings differ from the association with immune checkpoint inhibitors remains to be addressed in future studies.

## In Brief

This study investigated the effects of the early post-stroke application of cathodal transcranial direct current stimulation on the performance of the paretic upper limb (Fugl-Meyer Assessment scores) and the ROI-to-ROI correlations between motor areas. The findings suggest that increased connectivitymay not translate into clinical benefits promptly after a stroke.

## Highlights

Differences ≥9 points in Fugl–Meyer Assessment scores: sham>active.

Connectivity measures: active>sham.

## Graphical abstract

**Figure:**
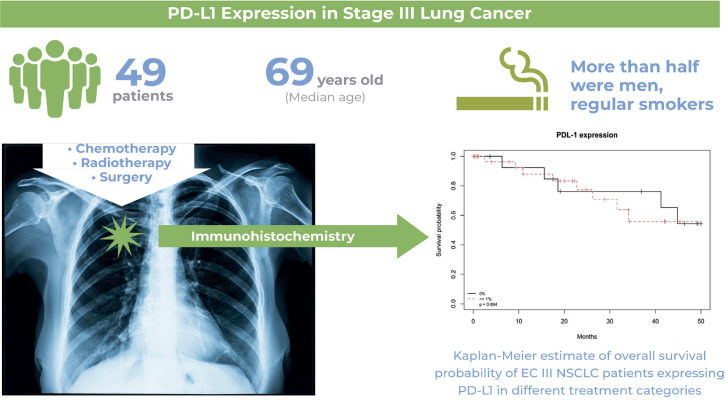

## INTRODUCTION

Globally, lung cancer is the second most commonly diagnosed cancer. However, it is still the leading cause of cancer-related death, thereby making it a major health concern. ^(1)^ Non-small cell lung cancer (NSCLC) accounts for approximately 85% of all lung cancer types.^(2)^

Approximately 20% of patients with NSCLC are diagnosed at a locally advanced stage, mostly because of the presence of locoregional lymph node involvement, which is classified as clinical stage III (ECIII) according to the eighth edition of the Tumor, Node and Metastasis (TNM) classification for lung cancer (AJCC 8^th^ edition). These patients generally have a poor prognosis and a 5-year median overall survival (OS) of 36%, 26%, and 13% for EC IIIA, EC IIIB, and EC IIIC, respectively.^(3)^

The treatment of patients with EC III disease varies according to the potential resectability of the primary tumor. Most guidelines and experts in the field recommend a combination of local and systemic treatments as the first choice, because of the potential risks of microscopic distant metastases.^(4)^ In general, surgical treatment followed by adjuvant therapy with platinum-based chemotherapy is recommended for patients with stage IIIA tumors, which are typically resectable.^(5)^ This therapy reduces the risk of mortality by approximately 5% over a 5-year period.^(6)^

Considering the limited efficacy of adjuvant treatments for resected lung cancer, multiple trials have investigated perioperative treatments with new molecules. Programmed cell death protein-1 (PD-1) and PD-Ligand (L) -1 have been the focus of several clinical investigations owing to their roles in the tumor microenvironment and as predictors of response to immune checkpoint inhibitors (ICI).^(7)^

The phase 3 IMPOWER010 trial showed a benefit in disease-free survival (DFS) by adding anti-PD-L1 atezolizumab for 1 year after adjuvant platinum-based chemotherapy in stage II-IIIA NSCLC patients, with a greater benefit in patients with PD-L1 expression ≥1%, with a gain in DFS (hazard ratio (HR)=0.66; 95% confidence interval (95%CI)=0.67-0.99, p=0.04) and a trend towards longer OS.^(8)^

For stage IIIB-IIIC patients whose tumor are considered unresectable, the standard treatment consists of a double platinum-based chemotherapy regimen combined with radiotherapy.^(9)^ However, despite all efforts, combined chemoradiotherapy for patients with poor prognoses can benefit only a small percentage of patients, with only 15% of patients surviving by the end of year 5.^(9,10)^ To achieve better outcomes, PD-L1 inhibitors are being evaluated with combined therapies to achieve higher responses.^(11)^ Chemoradiotherapy appears to upregulate PD-L1 and other immunogenic markers on the cell surface, thereby positively influencing a greater response to immunotherapy.^(12-14)^

In this unresectable subgroup of patients, a 1-year consolidation therapy with anti-PD-L1 durvalumab after chemoradiotherapy improved outcomes when compared to a placebo, in terms of progression-free survival (PFS), 16.9 versus 5.6 months (HR=0.55; 95% CI=0.45 to 0.68), and OS, 47.5 *versus* 29.1 months (HR=0.72; 95% CI=0.59 to 0.89).^(15)^ Overall, 42.9% of the patients survived in the durvalumab group versus 33.4% in the control group by year 5. Patients were selected and included in the PACIFIC trial, regardless of their PD-L1 expression level.^(15)^ The approval of durvalumab differs between regulatory agencies. It is approved by the Food and Drug Administration (FDA) and the Brazilian Health Regulatory Agency (ANVISA) regardless of PD-L1 expression, although the approval of durvalumad by the European Medicines Agency (EMA) is only for PD-L1 expression ≥1% based on post hoc analysis of the PACIFIC trial, which did not show an OS benefit for PD-L1 negative cases.^(16)^

Although the addition of PD-L1 inhibitors in the treatment of patients with stage III NSCLC has shown positive results; the effects of PD-L1 expression on stage III disease outcomes remain controversial.

## OBJECTIVE

This study aimed to evaluate PD-L1 expression as a predictor of progression-free survival and overall survival in patients with stage III (EC III) non-small cell lung cancer.

## METHODS

### Patients

Patients with EC-III NSCLC were evaluated at a tertiary hospital between January 2019 and January 2020. Data on patients’ sex and age, clinical and pathologic stage at diagnosis (8^th^ edition of the TNM staging system of lung cancer by AJCC/UICC), neoadjuvant and adjuvant treatment, surgery of the primary tumor, and PD-L1 expression were stratified by immunohistochemical (IHC) staining using a Dako Agilent PD-L1 IHC 22C3 kit (Agilent, Santa Clara, CA, USA). Individuals with incomplete data were excluded.

For resected cases, pathological reports were used for TNM staging, and clinical staging for unresectable case was defined using images. Overall survival was determined as the period between diagnosis and the date of death. Progression-free survival was defined as the time a patient survived during and after treatment without evidence of disease progression or death.

Optimal treatments for EC III lung cancer were based on the current National Comprehensive Network Guidelines 2023 (NCCN).^(17)^

The research ethics committee of *Hospital Israelita Albert Einstein* approved the study, which followed the existing national standards (CAAE: 81744017.6.0000.0071; #2.489.784). All datasets on which the conclusions of the report rely are available upon reasonable request to the corresponding author. The requirement for patient consent was waived due to the retrospective nature of the study.

### Statistical analyses

Quantitative variables are described as means and standard deviations or medians and interquartile ranges (IQR=1^st^ and 3^rd^ quartiles). Qualitative variables are described as absolute and relative frequencies.^(18)^ To evaluate the behavior of progression over time in the categories of variables of interest, cumulative incidence functions and nonparametric gray test graphs were constructed.^(19)^ The graphs present different curves according to the event and category, where the steps indicate the occurrence of the respective event.

To measure the risk of progression for each explanatory variable, including the quantitative variables, fine-gray survival models for competitive risks were used.^(20)^ The analysis of time to death and possible factors associated with the occurrence of this outcome were evaluated by simple Cox proportional hazards models. The assumption of risk proportionality was tested using Schoenfeld residuals.^(21)^ Analyses were performed using R.^(22)^ The cmprsk package was used for survival analyses. P<0.05 was considered statistically significant.

## RESULTS

A total of 49 patients with EC III NSCLC was included in this retrospective study. The clinical demographics of all the patients are shown in table 1. The median age of the overall population was 69 years (range: 53-85 years). More than half (65%) of the patients were men, and approximately 75% of the patients were regular smokers. Of the patients, 24% were treated with neoadjuvant chemotherapy. The regimens most commonly used in this setting include platinum salts (cisplatin or carboplatin) associated with paclitaxel, gemcitabine, or pemetrexed. Majority of the patients were treated with surgery (38%) or chemotherapy and radiotherapy (28%), sequentially or in combination. The regimens used in the combination included platinum salts (cisplatin or carboplatin), paclitaxel, or etoposide. The median PFS and OS of the cohort was 14.2 and 20 months, respectively (Table 2).

**Table 1:** Clinical characteristics of the patients in the cohort with EC III NSCLC

	Total patients (n=49)
Sex, n (%)	
Female	17 (34.7)
Male	32 (65.3)
Histology, n (%)	
Squamous	17 (34.7)
Adenocarcinoma	30 (61.2)
Non-Specified (NOE)	1 (2.0)
Adenosquamous	1 (2.0)
Stage, n (%)	
IIIA	21 (42.9)
IIIB	5 (10.2)
III (Non-Specified)	23 (46.9)
Smoking, n (%)	
No	12 (24.5)
Yes	37 (75.5)
Age at diagnosis (years)	
Median (SD)	69.8 (7.7)
Min-Max (n)	53.2-85.8 (49)
Pneumonitis, n (%)	
No	42 (85.7)
Yes	2 (4.1)
Related to radiotherapy	5 (10.2)
PD-L1, n (%)	
0	15 (30.6)
≥1	34 (69.4)
PD-L1, n (%)	
0	15 (30.6)
1-49	24 (49.0)
≥50	10 (20.4)
PD-L1, n (%)	
0-49	39 (79.6)
≥50	10 (20.4)

**Table 2:** Treatment types and outcomes of the patients

Neoadjuvant chemotherapy	
No	37 (75.5)
Yes	12 (24.5)
Neoadjuvant chemotherapy regimens	
Carboplatin + paclitaxel	3 (25.0)
Cisplatin + gemcitabine	3 (25.0)
Carboplatin + gemcitabine	2 (16.7)
Carboplatin + pemetrexed	3 (25.0)
Cisplatin+ pemetrexed	1 (8.3)
Definitive treatment	
Surgery	19 (38.7)
Chemotherapy combined with RDT	12 (24.5)
Chemotherapy followed by RDT	2 (4.0)
RDT	2 (4.0)
Chemotherapy	2 (4.0)
No treatment	12 (24.5)
Chemotherapy combined with RDT	
Cisplatin + RDT	2 (16.7)
Carboplatin + paclitaxel + RDT	4 (33.3)
Carboplatin + etoposide + RDT	1 (8.3)
Cisplatin + etoposide + RDT	4 (33.3)
Cisplatin + pemetrexed + RDT	1 (8.3)
Adjuvant regimens	
Chemotherapy	3 (6.1)
Radiotherapy	6 (12.2)
Chemotherapy combined with RDT	2 (4.1)
Chemotherapy followed by RDT	3 (6.1)
Adjuvant chemotherapy regimens	
Carboplatin + pemetrexed	3 (37.5)
Cisplatin + pemetrexed	3 (37.5)
Carboplatin + paclitaxel	1 (12.5)
Cisplatin + vinorelbine	1 (12.5)
Mortality	
No	35 (71.4)
Yes	14 (28.6)
Progression or death	
No	23 (46.9)
Progression	21 (42.9)
Death	5 (10.2)
Progression-free survival (months)	
Median [1º; 3º quartiles]	14.2 [4.6; 24.8]
Min-Max (n)	0.03-72.8 (49)
Overall survival (months)	
Median [1º; 3º quartiles]	20.1 [7.9; 41.2]
Min-Max (n)	0.03-120.9 (49)

RDT: radiotherapy.

### Disease progression and PD-L1 expression

**Figure 1:**
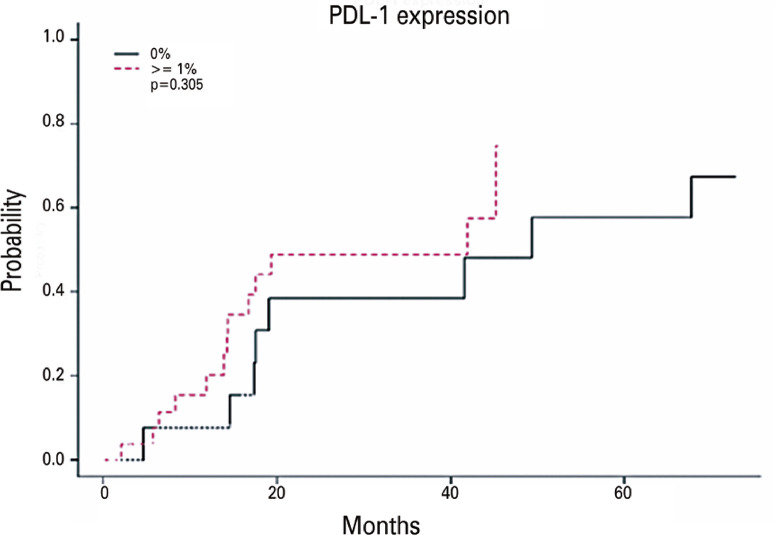
Adjusted Cox model on the probability of progression in relation to PD-L1 expression in all three groups

Descriptions of the patient characteristics based on occurrence of progression are presented in tables 1S and 2S, Supplementary Material. Among those with progression, 62% expressed PD-L1, and 14.3% had an expression ≥50%. Among those who did not show progression, 75% and 25% expressed PD-L1 and PD-L1 ≥50%, respectively. As shown in figure 1S, Supplementary Material the correlation between PFS and treatment type was not significant (p>0.05). No statistically significant association was identified between PD-L1 expression in the three categories (p>0.05; PD-L1 positive or negative, below, or above 50%; figure 1 and, Supplementary Material, figure 2S). There was no evidence of a significant association between PD-L1 expression in the categories observed in this study and disease progression, either when evaluated individually or per variable such as sex, disease stage, smoking status, neoadjuvant chemotherapy, or patient age (Table 3).

**Table 3:** Evaluation of variables associated with progression in each model

	Relative risk (95%CI)	p value
Simple Models for PD-L1 expression		
PD-L1		
0%	Reference	
≥1%	1.44 (0.62-3.34)	0.400
PD-L1		
0%	Reference	
1-49%	1.64 (0.64-4.24)	0.300
≥50%	1.02 (0.36-2.92)	0.970
PD-L1		
0-49%	Reference	
≥50%	0.79 (0.31-2.00)	0.620
Multiple Models: PD-L1 and sex		
PD-L1		
0%	Reference	
≥1%	1.33 (0.55-3.18)	0.530
Sex		
Female	Reference	
Male	0.76 (0.31-1.87)	0.550
Multiple Models: PD-L1 and Staging		
PD-L1		
0%	Reference	
≥1%	1.44 (0.68-3.04)	0.340
Staging		
IIIA	Reference	
IIIB	0.43 (0.10-1.90)	0.270
III (Non-specific)	0.42 (0.16-1.13)	0.087
Multiple Models: PD-L1 and smoking		
PD-L1		
0%	Reference	
≥1%	1.41 (0.61-3.26)	0.420
Smoking		
No	Reference	
Yes	0.85 (0.35-2.04)	0.710
Multiple Models: PD-L1 and neoadjuvant treatment		
PD-L1		
0%	Reference	
≥1%	1.50 (0.62-3.63)	0.370
Neoadjuvant Chemotherapy		
No	Reference	
Yes	1.26 (0.54-2.94)	0.600
Multiple Models: PD-L1 and age		
PD-L1		
0%	Reference	
≥1%	1.44 (0.62-3.34)	0.390
Age (years)	1.01 (0.96-1.08)	0.640

### Overall survival and PD-L1 expression

**Figure 2:**
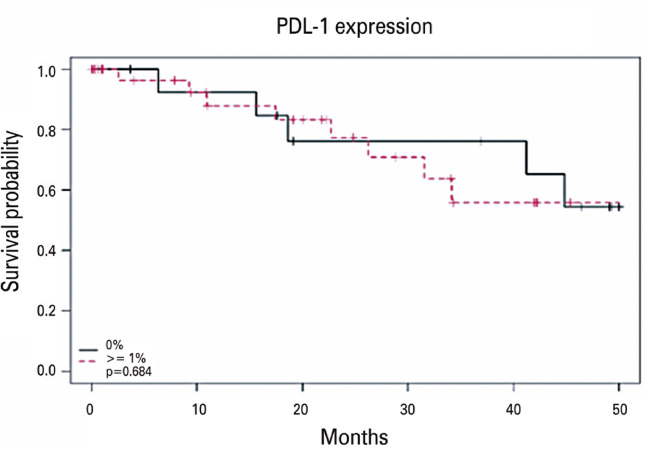
Kaplan-Meier estimate of overall survival probability of EC III NSCLC patients expressing PD-L1 in different treatment categories

Descriptions of the patient characteristics based on the occurrence of death are presented in tables 3S and 4S, Supplementary Material. Supplementary Material, figure 3S shows the correlation between the OS and treatment type. Among those who died, 64.3% had a positive expression of PD-L1, and 14.3% had an expression ≥50%. Among those who survived, 71.4% and 22.9% had a positive PD-L1 expression and an expression ≥50%, respectively. No statistically significant association was identified between PD-L1 expression in the three categories (p>0.05; PD-L1 positive or negative, below, or above 50%; Figure 2 and Supplementary Material, Figure 4S). No statistical difference (p>0.05) was observed for PD-L1 expression and risk of mortality, whether assessed independently or following adjustment for confounding variables such as sex, disease stage, smoking status, receipt of neoadjuvant chemotherapy, or patient age (Table 4).

**Table 4:** Evaluation of variables associated with overall survival of EC III NSCLC patients

	Relative risk (95%CI)	p value
Simple Models for PD-L1 expression		
PD-L1		
0%	Reference	
≥1%	1.26 (0.41-3.84)	0.684
PD-L1		
0%	Reference	
1-49%	1.28 (0.40-4.11)	0.678
≥50%	1.20 (0.22-6.42)	0.835
PD-L1		
0-49%	Reference	
≥50%	1.04 (0.22-4.83)	0.959
Multiple Models: PD-L1 and sex		
PD-L1		
0%	Reference	
≥1%	1.26 (0.41-3.84)	0.691
Sex		
Female	Reference	
Male	0.95 (0.31-2.93)	0.929
Multiple Models: PD-L1 and staging		
PD-L1		
0%	Reference	
≥1%	1.15 (0.35-3.85)	0.816
Staging		
IIIA	Reference	
IIIB	2.30 (0.35-15.01)	0.386
III (non-specific)	3.44 (0.92-12.86)	0.066
Multiple Models: PD-L1 and smoking		
PD-L1		
0%	Reference	
≥1%	1.52 (0.47-4.86)	0.482
Smoking		
No	Reference	
Yes	2.12 (0.54-8.38)	0.283
Multiple models: PD-L1 and neoadjuvant treatment		
PD-L1		
0%	Reference	
≥1%	1.13 (0.37-3.48)	0.828
Neoadjuvant chemotherapy		
No	Reference	
Yes	0.39 (0.09-1.77)	0.223
Multiple models: PD-L1 and age		
PD-L1		
0%	Reference	
≥1%	1.28 (0.42-3.89)	0.664
Age (years)	0.98 (0.91-1.05)	0.536

## DISCUSSION

PD-L1 expression has been a topic of interest in the treatment of NSCLC as it is a potential biomarker for predicting ICI treatment responses. However, the importance of PD-L1 expression for predicting treatment outcomes remain controversial. This retrospective study aimed to evaluate the effect of PD-L1 expression on PFS and OS in patients with EC III NSCLC using multiple treatment strategies. The analysis demonstrated that PD-L1 expression, as determined by IHC, was not statistically significant in predicting better outcomes.

Currently, PD-L1 expression is still the standard biomarker of response to ICI in NSCLC. For patients with stage II-IIIA NSCLC (UICC/AJCC staging system, 7^th^ ed) who received adjuvant platinum-based chemotherapy, without Epidermal Growth Factor Receptor (EGFR) mutation, adjuvant atezolizumab improved DFS (HR= 0·79; 0·64-0·96; p=0·020) for 16 cycles or 1 year. However, a greater benefit was identified in patients in the subgroup with a PD-L1 expression ≥1% (HR= 0.66; 95%CI= 0.67-0.99, p<0.05). After a median follow-up of 46 months, a trend toward improved OS was observed with atezolizumab.^(8)^

The Keynote 024 trial also demonstrated the benefit of anti-PD1 therapy in a population with PD-L1 positive expression.^(23)^ In the group of patients with PD-L1 ≥50%, better PFS (HR= 0.50; 95%CI= 0.37-0.68, p<0.001) and OS (HR= 0.60; 95%CI= 0.41-0.89; p<0.05) was achieved using first-line pembrolizumab.^(23)^ It is important to note that this trial included patients with advanced and metastatic disease.^(23)^ More recently, the EMPOWER-Lung 1 study conducted in 2021 also demonstrated an OS improvement (HR= 0.57; 95%CI= 0.42-0.77; p<0.05) with cemiplimab as first-line treatment for NSCLC with PD-L1 expression ≥50%.^(24)^

However, certain limitations related to PD-L1 expression need to be evaluated and addressed. First, the subjectivity and variability of the test kits are discussed.^(25)^ Investigation of PD-L1 expression is usually performed using IHC; however, some variation depending on the antibody is expected. For example, for patients receiving pembrolizumab-containing regimens, PD-L1 expression should be assessed using the 22C3 antibody, whereas the SP263 antibody can be used for patients receiving atezolizumab, and the 28-8 antibody for those receiving nivolumab.^(25)^ It is critical to note that these different antibody clones can produce variable results; this highlights the importance of proper validation of the assay methodology to ensure reliability as there are no standard methods available.^(25)^

Second, there was significant heterogeneity in intra-tumoral and inter-tumoral PD-L1 expression, which differed significantly according to the biopsy site.^(26)^ PD-L1 expression status can be substantially influenced by the sampling method (biopsy versus surgical resection), or even between the primary and metastatic site, which is known as spatial heterogeneity.^(26)^ Finally, PD-L1 expression can differ considerably depending on the assay used, with variable agreement in the same evaluated sample, even when considering expert pathologists.^(27)^ All these limitations could have influenced the findings of studies that evaluated PD-L1 expression in NSCLC.

In a meta-analysis of nine studies involving more than 1500 NSCLC patients, it was shown that high PD-L1 expression was solely associated with poor tumor differentiation (OR= 0.53; 95%CI= 0.39-0.72, p<0.0001).^(28)^ These data were somewhat corroborated by two other meta-analysis, which also showed an association of PD-L1 positivity with shorter OR (HR= 1.43; 95%CI= 1.24-1.63, p=0.329; HR= 1.75; 95%CI= 1.40-2.20, p<0.001).^(29,30)^ Conversely, Velcheti et al., identified a group of patients with an inflammatory tumor microenvironment by assessing PD-L1, demonstrating that this group of patients were related to a better prognosis.^(31)^ It should be noted that in this study, the correlation between PD-L1 positivity and tumor microenvironment was not assessed; however, there was no evidence of a significant relationship between PD-L1 expression and risk of mortality in patients with EC III NSCLC.

Furthermore, there was no evidence of an association between PD-L1 expression and better outcomes when evaluating multiple clinical factors such as sex, disease stage, smoking status, neoadjuvant chemotherapy type, or patient age. It is important to note that this study mainly included patients with adenocarcinoma, which may have influenced the results. In another retrospective analysis, a stronger correlation was observed between OS and PD-L1 expression in a group of lung squamous cell carcinomas (SqCLC).^(32)^ The correlation of PD-L1 expression and adjuvant therapy, increased tumor size (pT2-4), and positive lymph node status (pN1-3) has also been suggested.^(32)^

The effects of anti-PD-L1 treatments should also be considered. A retrospective analysis of 52 patients with stage III NSCLC treated with chemoradiotherapy followed by maintenance with durvalumab, like in the PACIFIC trial, showed that patients with PD-L1 expression ≥50% had a lower chance of disease progression and a better OS.^(33)^

Finally, PD-L1 expression may have been affected by the prior therapy. Some studies have indicated that neoadjuvant chemotherapy and EGFR-TKIs may decrease the expression of PD-L1.^(34,35)^ Additionally, some studies have demonstrated an increase in PD-L1 expression after chemotherapy, particularly with platinum-based regimens and radiotherapy.^(36,37)^ This suggests that PD-L1 expression may need to be reevaluated after therapy.

This study had some limitations that should be acknowledged. This was a retrospective study, and the sample size of the patients was relatively small; therefore, the grouped data could be below the statistical power. None of the patients received combined immunotherapy as the initial treatment; this may have influenced the results of this study. The strengths of this study include the importance and relevance of the biomarker PD-L1, considering multiple regimens that are being evaluated in clinical trials and a fairly large number of patients treated with multiple different regimens that will be mostly approximated with real-world clinical practice.

The datasets generated and/or analyzed in the current study are not publicly available because of the National General Data Law Protection (LGPD). These data are available from the corresponding author upon request.

## CONCLUSION

In this study, PD-L1 expression in stage III cell lung cancer was not correlated with any of the standard clinicopathological features, including sex, Tumor, Node and Metastasis stage, smoking status, neoadjuvant chemotherapy, or patient age, nor with disease outcomes. Further large-scale studies should be conducted to investigate the limitations and clinical and pathological importance of PD-L1 in stage III cell lung cancer.
