# Supplementary material for: Prognostic value of programmed cell death ligand 1 (PD-L1) expression in patients with stage III non-small cell lung cancer under different treatment types: a retrospective study
Source: Einstein (Sao Paulo). 2024 Jun 5;22:eAO0575. doi: 10.31744/einstein_journal/2024AO0575 (PMC11196088; doi:10.31744/einstein_journal/2024AO0575)
Supplement: Supplementary file 1 [file 2317-6385-eins-22-eAO0575-suppl01.pdf]

SUPPLEMENTARY MATERIAL

Prognostic value of programmed cell death ligand 1 (PD-L1) expression in patients with stage III non-small cell lung cancer under different treatment types: a retrospective study

Nicoly Marques de Castro, Fernando Moura, Aline Lury Hada, Diogo Garcia, Elivane da Silva Victor, Gustavo Schvartsman, Leonardo Carvalho, Milena Lourenço Coleta Fernandes, Rodrigo de Souza Martins, Elaine Ferreira da Silva, Sarah Silva Mello Batista dos Santos, Letícia Taniwaki, Patrícia Taranto, Janaina Pontes, Juliana Rodrigues Beal, Ana Carolina Pereira Dutra, João Bosco de Oliveira Filho, Sérgio Eduardo Alonso Araujo, Pedro Luiz Serrano Usón Junior

DOI: 10.31744/einstein_journal/2024AO0575

Table 1S. Clinical characteristics, PD-L1, and progression of EC III NSCLC patients

	Disease progression	
	No (n=28)	Yes (n=21)
Sex, n (%)		
Female	9 (32.1)	8 (38.1)
Male	19 (67.9)	13 (61.9)
Histology, n (%)		
Squamous	9 (32.1)	8 (38.1)
Adenocarcinoma	17 (60.7)	13 (61.9)
NOE	1 (3.6)	0 (0.0)
Adenosquamous	1 (3.6)	0 (0.0)
Staging, n (%)		
IIIA	10 (35.7)	11 (52.4)
IIIB	3 (10.7)	2 (9.5)
III (Non-specific)	15 (53.6)	8 (38.1)
Smoking, n (%)		
No	6 (21.4)	6 (28.6)
Yes	22 (78.6)	15 (71.4)
Age (years)		
Median (SD)	70.9 (7.7)	68.3 (7.7)
Min-Max (n)	53.2-85.8 (28)	54.2-81.7 (21)
Pneumonitis, n (%)		
No	24 (85.7)	18 (85.7)
Yes	1 (3.6)	1 (4.8)
Related to RDT	3 (10.7)	2 (9.5)
PD-L1, n (%)		
0%	7 (25.0)	8 (38.1)
≥1%	21 (75.0)	13 (61.9)
PD-L1, n (%)		
0%	7 (25.0)	8 (38.1)
1-49%	14 (50.0)	10 (47.6)
≥50%	7 (25.0)	3 (14.3)
PD-L1, n (%)		
0-49%	21 (75.0)	18 (85.7)
≥50%	7 (25.0)	3 (14.3)

EC III NSCLC: stage III non-small cell lung cancer; NOE: non-specified; SD: standard deviation; RDT: radiotherapy.

Table 2S. Description of treatments and outcomes according to tumor progression in PD-L1 expressing EC III NSCLC patients

	Disease progression	
	No (n=28) n (%)	Yes (n=21) n (%)
Neoadjuvant chemotherapy		
No	23 (82.1)	14 (66.7)
Yes	5 (17.9)	7 (33.3)
Neoadjuvant chemotherapy regimens		
Carboplatin+ paclitaxel	2 (40.0)	1 (14.3)
Cisplatin + gemcitabine	0 (0.0)	3 (42.9)
Carboplatin + gemcitabine	0 (0.0)	2 (28.6)
Carboplatin + pemetrexed	3 (60.0)	0 (0.0)
Cisplatin + pemetrexed	0 (0.0)	1 (14.3)
Definitive treatment		
Surgery	11 (39.3)	8 (38.1)
Chemoradiotherapy	4 (14.3)	8 (38.1)
Chemotherapy followed by RDT	1 (3.6)	1 (4.7)
RDT	1 (3.6)	1 (4.7)
Chemotherapy	0 (0.0)	2 (9.5)
Chemoradiotherapy		
Cisplatin + RDT	1 (25.0)	1 (12.5)
Carboplatin + paclitaxel + RDT	2 (50.0)	2 (25.0)
Carboplatin + etoposide + RDT	0 (0.0)	1 (12.5)
Cisplatin + etoposide + RDT	1 (25.0)	3 (37.5)
Cisplatin+ pemetrexed + RDT	0 (0.0)	1 (12.5)
Adjuvant treatment		
Chemotherapy	2 (7.1)	1 (4.8)
RDT	5 (17.9)	1 (4.8)
Chemoradiotherapy	2 (7.1)	0 (0.0)
Chemotherapy followed RDT	0 (0.0)	3 (14.3)
Adjuvant chemotherapy		
Carboplatin + pemetrexed	2 (50.0)	1 (25.0)
Cisplatin + pemetrexed	1 (25.0)	2 (50.0)
Carboplatin + paclitaxel	1 (25.0)	0 (0.0)
Cisplatin + vinorelbine	0 (0.0)	1 (25.0)
Deaths		
No	23 (82.1)	12 (57.1)
Yes	5 (17.9)	9 (42.9)

EC III NSCLC: stage III non-small cell lung cancer; RDT: radiotherapy.

Table 3S. Clinical characteristics, PD-L1 expression level, and mortality rate of EC III NSCLC[1] patients

	Mortality	
	No (n=35)	Yes (n=14)
Sex, n (%)		
Female	12 (34.3)	5 (35.7)
Male	23 (65.7)	9 (64.3)
Histology, n (%)		
Squamous	12 (34.3)	5 (35.7)
Adenocarcinoma	21 (60.0)	9 (64.3)
NOE	1 (2.9)	0 (0.0)
Adenosquamous	1 (2.9)	0 (0.0)
Staging, n (%)		
IIIA	18 (51.4)	3 (21.4)
IIIB	3 (8.6)	2 (14.3)
III (Non-specific)	14 (40.0)	9 (64.3)
Smoking, n (%)		
No	9 (25.7)	3 (21.4)
Yes	26 (74.3)	11 (78.6)
Age (years)		
Median (SD)	70.9 (7.2)	67.2 (8.7)
Min-Max (n)	53.2-85.8 (35)	54.2- 81.7 (14)
Pneumonitis, n (%)		
No	30 (85.7)	12 (85.7)
Yes	0 (0.0)	2 (14.3)
Related to RDT	5 (14.3)	0 (0.0)
PD-L1, n (%)		
0%	10 (28.6)	5 (35.7)
≥1%	25 (71.4)	9 (64.3)
PD-L1, n (%)		
0%	10 (28.6)	5 (35.7)
1-49%	17 (48.6)	7 (50.0)
≥50%	8 (22.9)	2 (14.3)
PD-L1, n (%)		
0-49%	27 (77.1)	12 (85.7)
≥50%	8 (22.9)	2 (14.3)

EC III NSCLC: stage III non-small cell lung cancer; NOE: non-specified; SD: standard deviation; RDT: radiotherapy.

Table 4S. Characterization of treatment modalities and outcome patterns relative to mortality

	Mortality	
	**No (n=35) n (%)**	**Yes (n=14) n (%)**
Neoadjuvant chemotherapy		
No	25 (71.4)	12 (85.7)
Yes	10 (28.6)	2 (14.3)
Neoadjuvant chemotherapy regimens		
Carboplatin+ paclitaxel	3 (30.0)	0 (0.0)
Cisplatin + gemcitabine	3 (30.0)	0 (0.0)
Carboplatin + gemcitabine	1 (10.0)	1 (50.0)
Carboplatin + pemetrexed	3 (30.0)	0 (0.0)
Cisplatin + pemetrexed	0 (0.0)	1 (50.0)
Definitive treatment		
Surgery	13 (37.1)	6 (42.9)
Chemoradiotherapy	9 (25.7)	3 (21.4)
Chemoradiotherapy	2 (5.7)	0 (0.0)
RDT1	0 (0.0)	2 (14.2)
Chemotherapy	1 (2.8)	1 (7.1)
Chemoradiotherapy		
Cisplatin + RDT	2 (22.2)	0 (0.0)
Carboplatin + paclitaxel + RDT	3 (33.3)	1 (33.3)
Carboplatin + etoposide + RDT	1 (11.1)	0 (0.0)
Cisplatin + etoposide + RDT	2 (22.2)	2 (66.7)
Cisplatin+ pemetrexed + RDT	1 (11.1)	0 (0.0)
Adjuvant treatment		
Chemotherapy	3 (8.6)	0 (0.0)
RDT	5 (14.3)	1 (7.1)
Chemoradiotherapy	1 (2.9)	1 (7.1)
Chemotherapy followed by RDT	1 (2.9)	2 (14.3)
Adjuvant chemotherapy		
Carboplatin + pemetrexed	1 (20.0)	2 (66.7)
Cisplatin + pemetrexed	2 (40.0)	1 (33.3)
Carboplatin + paclitaxel	1 (20.0)	0 (0.0)
Cisplatin + vinorelbine	1 (20.0)	0 (0.0)

RDT: radiotherapy.

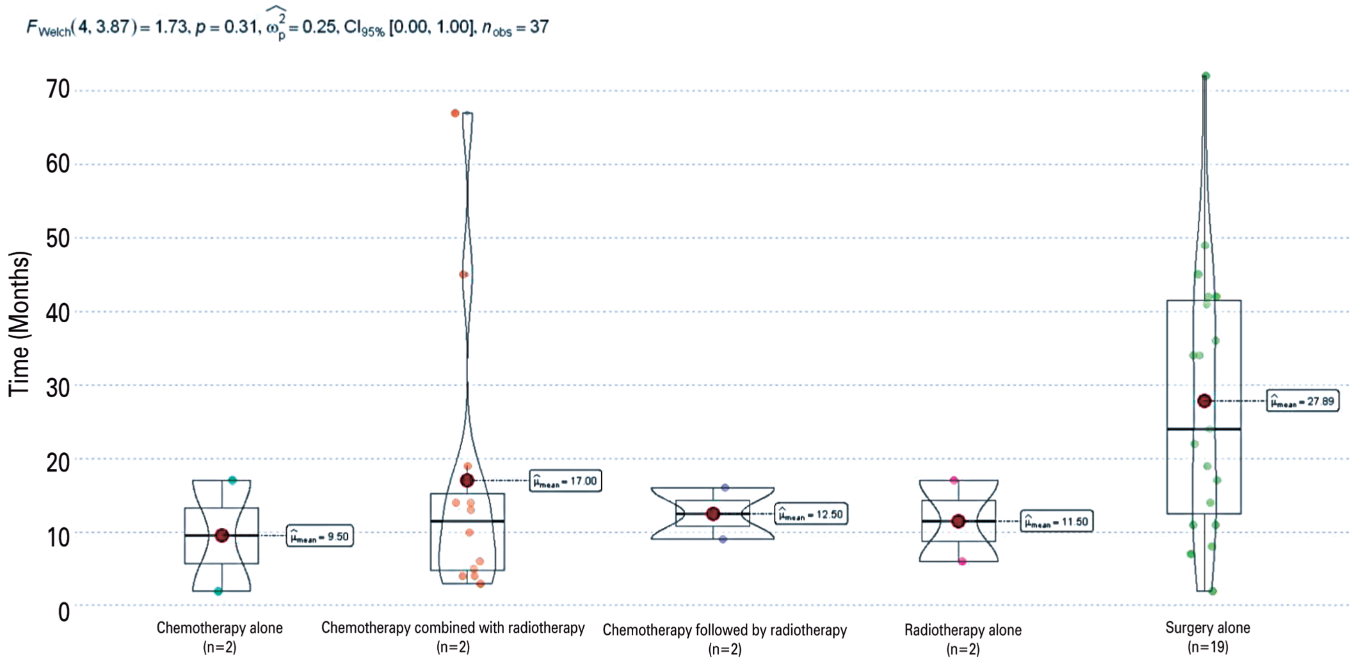

Figure 1S. Box and violin plots for the visualization for the progression-free survival (months) by the different cancer treatment types

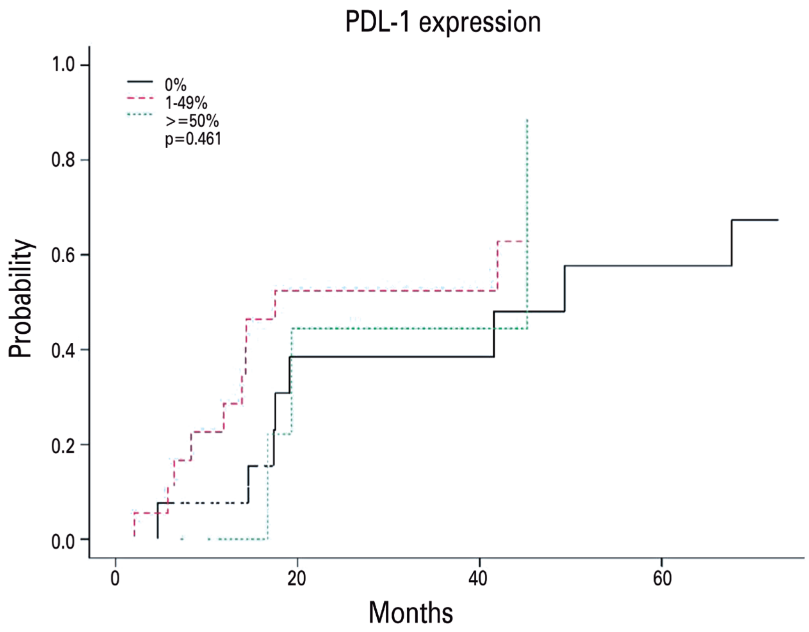

Figure 2S. Probability of Progression regarding PD-L1 expression in the three groups

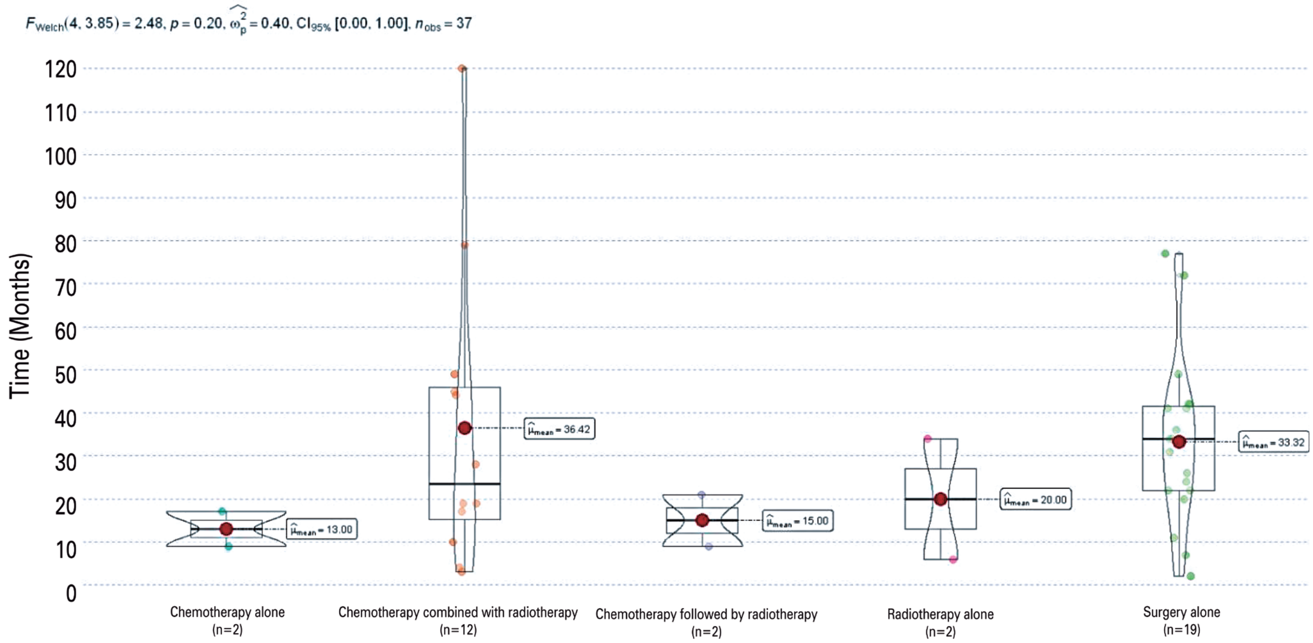

Figure 3S. Box and violin plots demonstrating the overall survival rate by the different treatment types

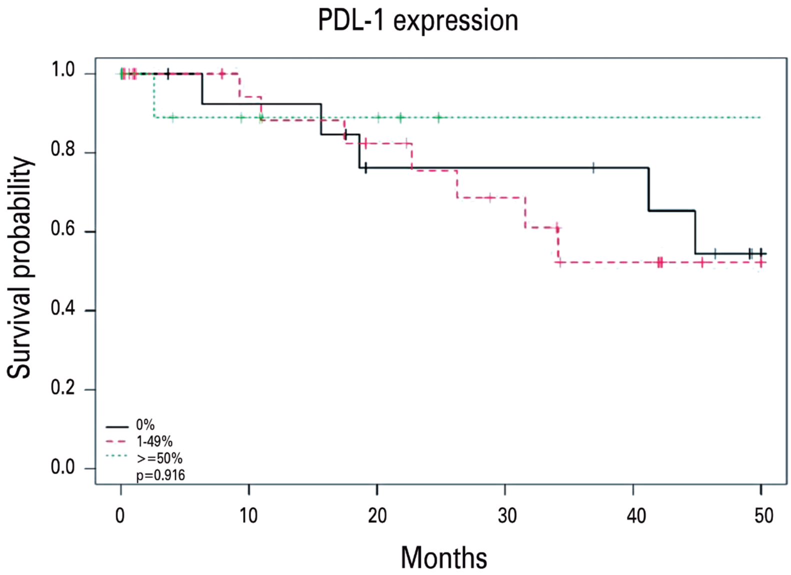

Figure 4S. Overall survival probability regarding the PD-L1 expression in the three groups
